# Binaural Fusion Sharpens on a Scale of Octaves During Pre-adolescence in Children with Normal Hearing, Hearing Aids, and Bimodal Cochlear Implants, but not Bilateral Cochlear Implants

**DOI:** 10.1007/s10162-025-00975-4

**Published:** 2025-02-06

**Authors:** Lina A. J. Reiss, Alicia J. Johnson, Morgan S. Eddolls, Curtis L. Hartling, Jennifer R. Fowler, Gemaine N. Stark, Bess Glickman, Holden Sanders, Yonghee Oh

**Affiliations:** 1 Department of Otolaryngology, Oregon Hearing Research Center, Oregon Health and Science University, 3181 SW Sam Jackson Park Road, Portland, OR 97239 USA; 2 Biostatistics and Design Program, Oregon Health and Science University, 3181 SW Sam Jackson Park Road, Portland, OR 97239 USA

**Keywords:** Binaural fusion, Pitch discrimination, Cochlear implant, Hearing aid, Development, Children

## Abstract

**Purpose:**

The breadth of binaural pitch fusion, the integration of sounds differing in frequency across the two ears, can limit the ability to segregate and understand speech in background noise. Binaural pitch fusion is one type of central auditory processing that may still be developing in the pre-adolescent age range. In addition, children with hearing loss potentially have different trajectories of development of central auditory processing compared to their normal-hearing (NH) peers, due to disruption of auditory input and/or abnormal stimulation from hearing devices. The goal of this study was to measure and compare binaural pitch fusion changes during development in children with NH versus hearing loss and different hearing device combinations. Interaural pitch discrimination abilities were also measured to control for pitch discrimination as a potential limiting factor for fusion that may also change during development.

**Methods:**

Baseline measurements of binaural pitch fusion and interaural pitch discrimination were conducted in a total of 62 (22 female) children with NH (*n* = 25), bilateral hearing aids (HA; *n* = 10, bimodal cochlear implants (CI; *n* = 9), and bilateral CIs (*n* = 18), with longitudinal follow-up for a subset of participants (18 NH, 9 HA, 8 bimodal CI, and 15 bilateral CI). Age at the start of testing ranged from 6 to 10 years old, with a goal of repeated measurements over 3–6 years. Binaural pitch fusion ranges were measured as the range of acoustic frequencies (electrodes) presented to one ear that was perceptually fused with a single reference frequency (electrode) presented simultaneously to the other ear. Similarly, interaural pitch discrimination was measured as the range of frequencies (electrodes) that could not be consistently ranked in pitch compared to a single reference frequency (electrode) under sequential presentation to opposite ears.

**Results:**

Children with NH and HAs initially had broad binaural pitch fusion ranges compared to adults. With increasing age, the binaural fusion range narrowed by 1–3 octaves for children with NH, bilateral HAs, and bimodal CIs, but not for children with bilateral CIs. Interaural pitch discrimination showed no changes with age, though differences in discrimination ability were seen across groups.

**Conclusion:**

Binaural fusion sharpens significantly on the scale of octaves in the age range from 6 to 14 years. The lack of change in interaural pitch discrimination with increasing age rules out discrimination changes as an explanation for the binaural fusion range changes. The differences in the trajectory of binaural fusion changes across groups indicate the importance of hearing device combination for the development of binaural processing abilities in children with hearing loss, with implications for addressing challenges with speech perception in noise. Together, the results suggest that pruning of binaural connections is still occurring and likely guided by hearing experience during childhood development.

**Supplementary Information:**

The online version contains supplementary material available at 10.1007/s10162-025-00975-4.

## Introduction

In children between 6 and 14 years old, central auditory processing abilities may still be developing. Certainly, monaural auditory processing measures such as frequency discrimination, amplitude modulation detection, and temporal integration have been shown to improve between 6 and 11 years of age [1, 2]. However, little is known about how factors such as disruption of auditory input with hearing loss, and/or abnormal patterns of stimulation from hearing aids (HAs) or cochlear implants (CIs), alter these developmental trajectories. One area of central auditory processing that may be affected by these factors during development is binaural fusion.

Binaural fusion is the perceptual integration or grouping of sounds received across the two ears into a single auditory object [3, 4]. The converse, binaural fission is the segregation of multiple sounds into separate auditory objects. The ability to both group and segregate acoustic components in an auditory scene is critical for the ability to separate out sounds in noise, such as a single talker from a multitude of talkers in the cocktail party effect [5–8]. However, recent findings showed that listeners with hearing loss often have abnormally broad binaural fusion, particularly of pitch. Whereas normal-hearing (NH) adults typically only fuse tones presented to opposite ears of similar frequencies less than 0.1 to 0.2 octaves apart, adults with HAs, bimodal CI (CI worn with HA in the contralateral ear), or bilateral CIs fuse sounds differing in pitch by up to 3 to 4 octaves [9–13]. This broad fusion occurs even with good sequential electrode discrimination or frequency discrimination on the order of 0.04–0.36 octaves, ruling out poor within-ear frequency resolution as an explanation for broad binaural pitch fusion [14]. Further, broader fusion is associated with greater difficulties with speech perception in background talkers [15, 16], likely due to fusion and averaging of speech even from talkers of different fundamental frequencies across ears [17, 18].

Recently, it was shown that both NH children and children who use bilateral HAs between ages 6 and 11 have broader binaural fusion than their equivalent adult counterparts [19], suggesting that binaural fusion may still be developing (sharpening) in this age range. Interestingly, that same study showed that children with bimodal or bilateral CIs did not differ significantly in binaural fusion range from their equivalent adult counterparts, though the bilateral CI group had great variability with some children exhibiting no fusion and others exhibiting very broad fusion. The bilateral CI group may differ from the other groups in the lack of early acoustic experience, which may be necessary for the initial development of inter-hemispheric connections. Certainly, early acoustic experience has been shown to be critical for the development of tonotopic maps in the brain [20]; some tonotopy can be restored with CI stimulation as early as 10 weeks of age but not after 8 months [21, 22]. Hence, early acoustic experience may also be necessary for the initial formation of broad interaural connections for binaural fusion.

Another factor of interest is interaural pitch discrimination, in which stimuli are presented sequentially instead of simultaneously. The slope or bandwidth of the interaural pitch match function can be considered a measure of interaural pitch discriminability. The slope will be shallower and the range broader if there is more overlap in neural populations being activated and compared across the ears. A broad pitch match bandwidth may explain broad binaural fusion, and similarly changes in binaural fusion during development may be explained by changes in pitch match bandwidth, so this is a necessary control condition. Previous studies do show improvements in within-ear frequency discrimination over this age range between 6 and 11 years, e.g., in frequency discrimination thresholds [1, 23]. The developmental trajectory of interaural pitch discrimination abilities may also differ across groups.

There is limited knowledge of how binaural fusion develops in children and how the trajectory differs between NH children and children who use HAs and/or CIs. The goal of the current study was to follow children longitudinally over 3–6 years and measure how binaural pitch fusion and interaural pitch discrimination (1) change with age and (2) differ between groups with different hearing devices. We hypothesized that both binaural pitch fusion and interaural pitch discrimination would improve (sharpen) with age for children with NH, bilateral HAs, bimodal CIs, and bilateral CIs. A secondary hypothesis was that developmental trajectories would differ with hearing device combination, as each device combination produces exposure to different neural stimulation patterns during this stage of development.

## Methods

The methodology generally follows that of Hartling et al. [19] which reported the initial (first year) results of the study. Methods are recapped below with a focus on the longitudinal aspects; the earlier study can be referred to for more details.

### Subjects

A total of 62 children (mean baseline age, 7.4 ± 0.83 years) participated in the study, with 25 NH children, 10 bilateral HA users, 9 bimodal CI users, and 18 bilateral CI users, designated in subject identification labels as NK, HK, CK, and BK, respectively. After initial enrollment, a total of 50 children (mean baseline age, 7.7 ± 1.17 years), consisting of 18 NK, 9 HK, 8 CK, and 15 BK participants, continued longitudinal follow-up.

At the initial visit, all subjects were interviewed together with their parent or caregiver prior to testing to obtain information on age, gender, and number of ear infections. Hearing-impaired children were also asked about hearing loss history (etiology, onset, duration, and time course of hearing loss), hearing device type and history, and daily hours of hearing device use. Hearing loss history, hearing device history, and audiometric data were also collected from OHSU medical records for OHSU patients or obtained from audiology service providers for non-OHSU patients. In addition, to guide the selection of images used to keep children engaged during the experiment, children were asked about their favorite toys, games, movies, pets, sports, and other hobbies. Baseline age, sex, number of ear infections, and baseline years of hearing experience with each hearing device are summarized for those participants by group in Table 1, along with the age range of longitudinal follow-up. The number of ear infections at baseline was determined based on parent report throughout the study. Notably, two participants had early childhood ear infections reported at follow-up timepoints but not at baseline; these were included as infections in Table 1.

**Table 1:** Summary demographics of longitudinal follow-up participants in the four groups. Mean and standard deviation (SD) of age, number (*N*) and percentage (%) of each sex, *N* and % of ear infections, and mean and SD of years of hearing experience with the current hearing devices are given for each group. Note that for the CK group, the total duration of HA use in the non-CI ear is given, which can include both the duration of bilateral HA use before the CI and the duration of bimodal (combined CI + HA) use after the CI

	NK (*N* = 25)	HK (*N* = 10)	CK (*N* = 9)	BK (*N* = 18)
Baseline age, mean (SD)	7.4 (0.83)	7.7 (1.17)	8.0 (1.52)	7.7 (1.31)
Sex, *N* (%)				
Male	16 (64.0)	10 (100.0)	3 (33.3)	11 (61.1)
Female	9 (36.0)	0 (0.0)	6 (66.7)	7 (38.9)
Ear infections, *N* (%)				
None	14 (56.0)	6 (60.0)	2 (22.2)	11 (61.1)
One	2 (8.0)	1 (10.0)	0 (0.0)	1 (5.6)
> 1 or severe infection	9 (36.0)	3 (30.0)	7 (77.8)	6 (33.3)
Years of hearing experience, mean (SD)		L: 4.4 (2.97), R: 4.4 (2.97)	HA: 6.2 (2.93), CI: 4.6 (2.18)	L: 4.6 (2.44), R: 5.3 (2.07)

**Fig. 1:**
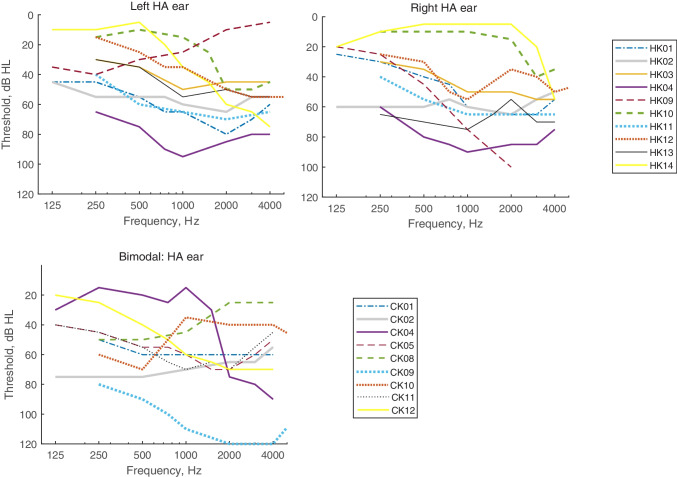
Audiometric thresholds for HK and CK participants. The top row shows the audiograms for the left and right ears of HK participants, and the bottom row shows the audiogram for the non-implanted ear of CK participants

Children with acoustic hearing underwent audiometric evaluation at the initial visit: otoscopy, tympanometry, and pure tone air and bone conduction thresholds; bone conduction thresholds were measured to rule out conductive losses. The NH children (NK group) were all confirmed to have audiometric thresholds ≤ 20 dB HL from 250 to 4000 Hz. Initial audiograms for the left and right ears of the bilateral HA (HK) group and the HA ear of the bimodal CI (CK) group are shown in Fig. 1. The hearing-impaired children all had at least 1 year of experience with their current combination of hearing devices. Tables 2, 3, and 4 show the ages, gender, etiology of hearing loss, duration of hearing loss, duration of use of each hearing device combination, daily hours of use of the current devices, device models, and reference ears for the HK, CK, and BK groups, respectively. Note that the duration of HA use for CK groups is reported differently in Tables 1 and 3, in that Table 1 shows HA use duration in the non-CI ear (including both pre- and post-CI) and Table 3 shows bilateral HA use duration before the CI. The average start and end age for the NK group was 7.4 ± 0.8 and 10.7 ± 2.8 years, respectively; the average start and end age for the other groups are shown in the respective tables. A one-way ANOVA showed no significant differences in baseline age across the four participant groups (*p* = 0.64).

**Table 2:** Demographic information for bilateral hearing aid (HA) pediatric subjects (HK): age range tested (age start and age end), gender, etiology of hearing loss, baseline duration of experience with HAs, daily hours of bilateral HA (BHA) use, HA make and model, and reference ear. Duration of HA experience and daily hours of HA use were the same for both ears for all participants, so only one number is shown. *HL*, hearing loss; *L*, left; *R*, right. *M*, male; *F*, female

Subj. ID	Age start (yrs)	Age end (yrs)	Sex	Etiology of HL	Dur. BHA use (yrs)	Daily HA use (hrs/day)	HA model	Ref. Ear
HK01	8.9	13.1	M	Unknown	7.4	10	Oticon Safari 300 BTE Power	L
HK02	6.5	13.1	M	Unknown	2.1	12	Oticon Safari 300 BTE Power	L
HK03	6.7	11.1	M	Unknown	1.9	13	Oticon Safari 300 BTE Power	L
HK04	6.3	12.6	M	Unknown	4.0	12	Phonak Naida IX SP	R
HK09	7.7	12.4	M	Underdeveloped cochlea	1.7	8	Phonak Sky Q50-SP	R
HK10	9.2	14.7	M	Unknown	2.0	9	Oticon Sensei BTE 75	L
HK11	7.5	10.5	M	Unknown	6.6	16	Oticon Sensei Pro BTE 90	R
HK12	7.4	7.4	M	Unknown	7.2	14	Oticon Safari 300 BTE	L
HK13	9.7	14.9	M	Unknown	9.4	14	Phonak Naida Q70-SP	R
HK14	7.6	11.4	M	Unknown	1.6	14	Phonak Sky Q70 M13	L
Avg	**7.7**	**12.1**			**4.4**	**12.2**		
Std	**1.2**	**2.2**			**3.0**	**2.5**

**Table 3:** Demographic information for CK subjects: age range tested, gender, etiology of hearing loss, baseline duration of experience with bilateral HAs before CI, baseline duration of CI experience, daily hours of listening in bimodal mode (BM; both CI and HA), HA make and model, CI internal device, and reference ear. *AB*, Advanced Bionics. *M*, male; *F*, female. Note that the duration of CI experience was the same as the duration of bimodal experience for all participants except for one participant, CK09, who did not wear their HA with the CI and was not truly bimodal

Subj. ID	Age start (yrs)	Age end (yrs)	Sex	Etiology of HL	Dur. BHA pre-CI (yrs)	Dur. CI (yrs)	Daily BM use (hrs/day)	HA model; CI internal device	Ref. (CI) Ear
CK01	7.1	12.9	M	Pendred	5.9	1.0	14	Phonak Naida; Cochlear CI512	R
CK02	7.8	11.9	F	Unknown	0.9	6.1	12	Widex Mind 440; Cochlear CI24RE	L
CK04	7.4	11.9	F	Unknown	1.0	1.2	13	Phonak Ambra; Cochlear CI422	R
CK05	6.8	11.1	F	Connexin 26	1.9	4.6	13	Widex Inteo; Cochlear CI512	L
CK08	9.9	12.3	M	Unknown progressive	4.6	4.2	14	Phonak Sky Q70-UP; Cochlear CI24RE	R
CK09	8.7	8.7	F	Connexin 26	1.3	7.0	0	Phonak Naida; CI512	R
CK10	7.3	8.4	F	Waarden-burg type 2	1.0	6.0	12	Phonak V70-P; MED-El Concert: Standard electrode	L
CK11	6.2	9.3	F	Unknown	1.0	4.5	12	Oticon Sensei Pro BTE 90; AB HiFocus MS	R
CK12	10.8	14.7	M	Unknown progressive	4.2	6.4	13	Oticon Sensei Pro; AB HiFocus 1 J	R
Avg	**8.0**		**11.3**		**2.4**	**4.6**	**11.4**		
Std	**1.5**		**2.1**		**1.9**	**2.2**	**4.4**

**Table 4:** Demographic information for BK subjects: age range tested, gender, etiology of hearing loss, baseline durations of experience with bilateral HAs before CI, bimodal or CI-only before the second CI, and bilateral CIs, daily hours of bilateral CI use, CI internal devices, and reference ear. Numbers separated by semicolons indicate values for the left; right ears if different for the two ears. CI internal devices are from Cochlear unless otherwise specified. *M*, male; *F*, female

Subj.ID	Age start (yrs)	Age end (yrs)	Sex	Etiology of HL	Dur. BHA pre-1^st^ CI (yrs)	Dur. BM pre-2^nd^ CI (yrs)	Dur. BiCI (yrs)	Daily biCI use (hrs/day)	CI internal devices	Ref. Ear
BK01	6.6	9.5	M	Unknown	0.75	0.5	5.1	12	CI24RE; CI512	R
BK02	7.4	10.6	F	Genetic	0.0	0.0	4.9	16	CI24RE; CI512	L
BK03	7.7	7.7	F	Connexin 26	0.0	0.0	5.9	13	CI24RE	R
BK04	6.1	6.1	F	EVA; malformed cochlea	0.25	0.5	4.7	13	MED-EL Sonata ti100: Standard	R
BK05	6.4	12.3	M	Pendred	1.4	1.5	1.0	14	CI422	R
BK06	8.5	14.2	F	Unknown	0.0	0.0	6.9	15	CI24RE	R
BK08	6.1	13.9	M	Genetic	0.25	0.0	4.9	13	CI512	L
BK09	8.6	11.7	M	Unknown progressive	1.2	3.5	1.3	12	CI24RE	R
BK10	10.0	12.1	M	Genetic progressive	2.6	4.8	1.1	13	CI512; CI422	L
BK11	9.1	9.1	M	Meningitis progressive	0.0	0.0	7.3	13	CI24RE	R
BK12	9.2	12.3	M	Unknown	0.0	3.0	1.8	14	CI512; CI24RE	R
BK14	7.3	9.4	M	Connexin 26 progressive	4.75	1.5	0.8	9	CI522; CI512	L
BK15	6.5	11.2	F	Myosin 15a progressive	1.1	0.8	2.6	13	CI422	R
BK16	6.6	11.1	F	Connexin 26	0.7	0.0	5.1	14	CI24RE	L
BK17	10.2	14.7	M	Connexin 26	0.6	0.3	9.3	14	CI24RE	L
BK18	7.7	12.0	M	Unknown	0.5	1.7	1.8	14	CI24RE	R
BK19	6.8	10.8	M	Usher Type 1	0.7	0.0	5.9	13	CI512	R
BK20	8.1	9.8	F	Connexin 26	0.42	0.3	7.3	15	CI512	L
Avg	**7.7**	**11.0**			**0.8**	**1.0**	**4.3**	**13.3**		
Std	**1.3**	**2.2**			**1.2**	**1.4**	**2.6**	**1.5**		

Subsequent follow-up visits were conducted approximately annually for up to 7 years, though due to COVID there were gaps in testing for some subjects. Questionnaires were re-administered annually to update information such as ear infections, hearing device type and history, daily hours of hearing device use, and interests. Audiometric tests were re-administered, and programming history was downloaded prior to each visit. Most subjects had less than 10 dB changes in audiometric thresholds over the duration of participation, with the exception of two bimodal CI subjects (CK01 and CK08) and three bilateral HA subjects (HK01, left ear; HK10, right ear; HK13, both ears). CK01, CK08, HK10, and HK13 had threshold shifts of ~ 20 dB, while HK01 had threshold shifts ranging from 20 to 40 dB between 1000 and 4000 Hz; this participant was also tested with a lower reference frequency in all experiments. All participants were paid for their time spent in the study, and travel and overnight accommodations were provided for those participants from out of town.

### General Procedures

All procedures and experiments were conducted in a double-walled, sound-attenuated booth. Stimuli were presented via computer to control electric and acoustic stimulus presentations. The NK and HK groups were tested using acoustic stimuli under headphones. For the CK group, electric stimuli were presented via an experimental CI processor to the implanted ear, and acoustic stimuli via headphone to the opposite ear. For the BK group, electric stimuli were presented to both ears. All subjects were tested unaided using headphones and/or experimental CI processors; no personal HAs or CI processors were used.

Acoustic stimuli were generated at a sampling rate of 44.1 kHz with MATLAB (version R2010b), generated by an ESI Juli sound card (for NK, HK, and CK subjects with Cochlear devices) or the PCIe-6351 card (for CK subjects with MED-El or Advanced Bionics devices), TDT PA5 digital attenuator and HB7 headphone buffer, and presented over Sennheiser HD-25 headphones. All acoustic stimuli consisted of sinusoidal pure tones with 10-ms raised-cosine onset/offset ramps. For acoustic loudness balancing, 300-ms tones at 0.125, 0.175, 0.25, 0.375, 0.5, 0.625, 0.75, 0.875, 1, 2, 3, and 4 kHz were used.

Electric stimuli were delivered to the CI using NIC2, RIB2, and BEDCS CI research software for Cochlear, MED-EL, and Advanced Bionics devices, respectively. The hardware consisted of clinical programming pods and L34 research processors for Cochlear and a National Instruments PCIe-6351 card for MED-EL and Advanced Bionics. Stimuli were synchronized across ears for CK and BK subjects using triggering via the trigger input feature of the programming pods for Cochlear and the PCIe-6351 card for MED-EL and Advanced Bionics. Pulse widths were those used in the subjects’ clinical programs. All CI subjects were stimulated using monopolar stimulation with a pulse rate of 1200 pps/electrode to minimize the effects of any temporal cues on pitch; all subjects and electrodes tested used clinical pulse rates of 900 pps or higher, also above this rate limit.

 All stimuli were loudness balanced using a method of adjustment. First, the level of the acoustic or electric stimulation for each stimulus was initialized to a comfortable level using a simplified visual loudness scale with three options: “too soft,” “good,” and “too loud.” For hearing-impaired acoustic ears, tones that could not be presented loud enough to reach a “good” level due to too much hearing loss at those frequencies were excluded; this determined the upper limits of the loudness-balanced frequency range of the acoustic ear. Second, once levels were initialized, all stimuli were loudness balanced both within and across ears, by presenting each pair of stimuli sequentially and adjusting levels until the two stimuli were judged to be similar in loudness. For acoustic stimuli, interpolation (on a dB and log frequency scale) was then used to determine appropriate levels for all usable tone frequencies within the 125–4000 Hz frequency range used for testing.

After the loudness balancing procedure, the same interface was used to assess the understanding of pitch. Two stimuli with large pitch differences were presented sequentially, and the child was asked to indicate verbally if the first or second stimulus was higher in pitch, and verbal feedback was given by the experimenter. A significant number of younger children, including NH children, did not understand the concept of pitch initially. In those cases, the concept was taught using various methods including a toy keyboard, ecological examples (animal sounds, male versus female voices), and musical activities (such as movement to visualize pitch). Conceptual understanding was verified using practice pitch matching tests conducted within ears, with feedback, and was repeated at each visit to ensure understanding of the concept of pitch was retained and to re-familiarize participants with listening for pitch cues.

Dichotic fusion range and interaural pitch matching measurements were conducted using the same reference ear and stimulus. For NK, HK, and BK subjects, the reference ear was chosen randomly for ears with symmetric pitch discrimination; otherwise, the reference ear was chosen to be the ear with worse pitch discrimination. For CK subjects, the reference ear was the CI ear. For NK and HK subjects, the reference stimulus was a 1.6-kHz tone because it is above the binaural beat frequency detection limit for NH listeners [24], to avoid binaural beats adding difficulty to the task. One participant, HK01, was tested with a 0.5-kHz reference stimulus and was verified as not hearing binaural beats at 0.5 kHz using an adaptive beat detection procedure [10, 25]. For CK and BK subjects using Cochlear devices, the reference stimulus was electrode 18 (frequency-to-electrode allocation range, 688–813 Hz), with the exception of CK01 which was tested with electrode 22 (frequency-to-electrode allocation range, 188–313 Hz) due to a limited acoustic hearing range. For CK and BK subjects using MED-EL and Advanced Bionics devices, the reference stimulus was electrode 5 which had similar frequency-to-electrode allocations and was not among the apical four electrodes used in MED-EL’s fine-structure strategy (MED-EL frequency range, 787–1144 or 935–1383 Hz; Advanced Bionics frequency range, 697–828 Hz).

Subjects were given practice trials with feedback before each procedure. Within each run of the fusion range or pitch matching task, the reference stimulus was fixed, and the comparison stimulus varied in pseudorandom sequence from trial to trial for a total of six repeat trials for each comparison stimulus per run [26]. Comparison stimuli in the contralateral ear were acoustic tones for NK, HK, and CK subjects, and electric pulse trains to individual electrodes in the contralateral CI for BK subjects. For BK subjects with MED-El, all 10–12 electrodes were used to deliver comparison stimuli. For BK subjects with Cochlear devices, which have 22 electrodes spaced at less than half the spacing of MED-EL electrodes, only even-numbered electrodes were tested in order to limit the number of trials. At least two repeat runs were performed for each reference electrode and procedure.

A variety of methods were used to keep children engaged and on task, especially in the first few years of the study. First, the successful completion of each trial was followed by the appearance of a third screen with an image in a 5 × 4 grid, with one random piece of the image revealed incrementally after each trial, so that the full image was displayed after every 20 trials. Images were chosen based on the child’s interests as determined in the initial questionnaire. Second, a second experimenter was available to assist in the booth to monitor the child, help them to stay engaged, provide small rewards such as stickers at intervals, and determine based on behavioral cues when a break was needed. Third, successful completion of each full procedure was followed by a larger reward such as a small toy or a store gift certificate to accumulate for larger prizes.

### Dichotic Fusion Range Measurements

**Fig. 2:**
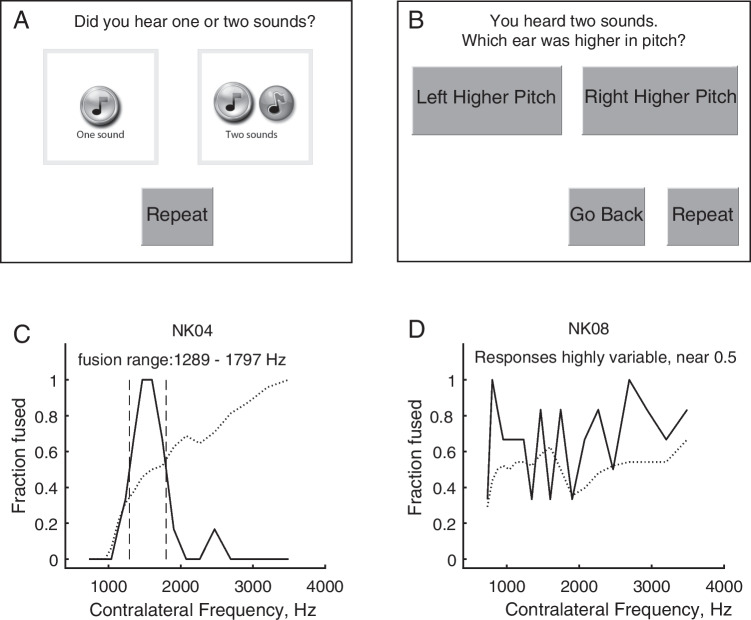
Fusion range task. **A** Touchscreen response options for the first question in the task, “Do you hear one or two sounds?” **B** Touchscreen response options for the second question in the task if two sounds were heard “Which ear was higher in pitch?”** C** Example of successful fusion range function (solid curve) with a clear frequency range of fusion consistently greater than 50%. Also shown is the pitch ranking function (dotted curve) when two sounds were heard, used as verification that subjects really heard two sounds and could determine which ear had the higher pitch, indicated by a monotonically increasing function. **D** Example of failure in fusion range task, with noisy or inconsistent responses. A and B are reprinted with permission from Ear and Hearing

The dichotic fusion range, or the range of comparison stimuli that were fused with the reference stimulus, was measured using a single interval procedure. For each trial, the comparison stimulus was presented *simultaneously* with the reference stimulus over a duration of 1500 ms. Subjects were asked to indicate on a touchscreen display whether they heard one or two sounds, by selecting one of two response buttons with one or two musical notes, respectively (Fig. 2A). If they heard only one sound, regardless of whether it was heard only in one ear or both ears, they were instructed to choose the “One Sound” button. If they heard different sounds in each ear, then they were instructed to choose the “Two Sounds” button. If the “Two Sounds” button was selected, then another screen appeared with two response buttons titled “Left Higher Pitch” and “Right Higher Pitch” (Fig. 2B). Subjects were asked to choose which ear they heard the higher pitched sound of the two sounds, by selecting the appropriate button. If subjects could not tell which was higher in pitch, they were instructed to guess and pick one of the two ears randomly. From this screen, subjects could also choose to “Go Back” if they changed their mind and wanted to choose “One Sound.” This second screen was provided as a check of whether the subject really heard two sounds and that they understood the task (for pitches that are sufficiently different to be correctly ranked in sequential stimulation, subjects should be able to identify which ear had the higher pitch). A “Repeat” button was also provided on both screens to allow subjects to listen to the stimuli again as often as needed.

For analysis, responses were assigned fusion values as follows: “Left Higher Pitch” or “Right Higher Pitch” = 0, and “One Sound” = 1. Each point on the fusion function was calculated as these fusion values averaged over all trials for each contralateral reference stimulus, and indicated the proportion of trials that the reference stimulus was fused with that contralateral stimulus as a function of the pair stimulus frequency or electrode. For each fusion function (example solid curve in Fig. 2C), the fusion range was defined as the frequency or electrode range between the 50% points (P(fused) = 0.5; demarcated by dashed vertical lines). The fusion range is the stimulus range (acoustic tone frequencies or CI electrodes) in one ear that fused with a single reference stimulus (acoustic tone frequency or CI electrode) in the other ear.

However, several subjects, particularly in the BK group, were not able to conduct the fusion range task, especially in the first few years, as indicated by noisy, nearly flat responses around 50%. To quantify the number of successes and failures, task completion for the fusion range task was defined as having a fusion range function with a consistent, measurable range of contralateral stimuli that fused with the reference stimulus more than 50% of the time (e.g., Fig. 2C). An additional form of verification was conducted when two sounds were heard: whether the subject was able to correctly rank which of the two sounds was higher in pitch (e.g., dotted curve in Fig. 2C). Correct pitch ranking was indicated when the percentage of time that the comparison stimulus was ranked higher increased progressively as the comparison stimulus became higher in frequency or more basal in electrode location compared to the reference, and accuracy was higher when the comparison was farther from the reference and outside the fusion range. Task completion failures (e.g., Fig. 2D) were excluded from further data analyses at those time points.

### Interaural Pitch Matching

**Fig. 3:**
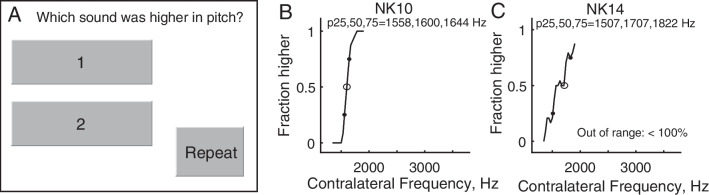
Pitch matching task. **A** Touchscreen response options.** B** Example of successful pitch match bracketed by 0% and 100% points. **C** Example of failure in pitch match function. “Out of range < 100%” indicates that the function never reached 100%

Prior to testing, subjects were trained using a within-ear pitch ranking test with feedback. Interaural pitch matches were then obtained using a two-interval, two-alternative forced-choice constant stimulus procedure. One interval contained a reference stimulus delivered to the reference ear. The other interval contained a comparison stimulus delivered to the contralateral ear; stimuli were either acoustic or electric, depending on the subject group. The stimuli were each 500 ms in duration and separated by a 500-ms interstimulus interval, with interval order randomized. In each trial, the subject was asked to indicate on a touchscreen display which interval had the higher pitch (Fig. 3A). Note that requiring subjects to identify the interval with the higher pitch tone is more difficult than simple discrimination, i.e., picking the different sounding stimulus in a 3- or 4-alternative forced-choice procedure. This more difficult type of task was chosen to avoid the use of other cues that could be used to pick the different sounds, such as loudness or other dimensions than pitch as can occur with cochlear implants [27].

Pitch match psychometric functions were computed as the averaged responses to the range of comparison stimuli (example shown as solid curve in Fig. 3B). When functions showed clear bracketing of the pitch match with *y*-values increasing from 0 to 1, pitch match centers were computed as the 50% points on the functions (P(higher) = 0.5; open circles), and pitch match ranges were computed as the distance between the 25 and 75% points (P(higher) = 0.25 and 0.75; filled circles) in the psychometric function. This pitch match range is used as a measure of slope, with narrow ranges indicating steep slopes and broad ranges indicating shallow slopes. Interestingly, the pitch match functions obtained using sequential stimulation here are much narrower, with steeper slopes, than those obtained using simultaneous stimulation in the fusion task (Supplementary Fig. 1). The shallower slopes of the simultaneous pitch match functions are not explained simply by increased fusion, as the poorer performance extends beyond the fusion region. This might indicate the greater difficulty of determining which tone was heard in which ear in the fusion task, or alternatively, possible instances of partial fusion, in which two tones are still heard, but are partially fused and partially averaged in pitch [28].

Most subjects were able to pitch match the reference stimuli (acoustic tones or CI electrodes) to the comparison stimuli in the contralateral ear. However, several subjects, particularly in the BK group, were not able to pitch match at all, especially in the first few years, as indicated by mostly flat or non-monotonic pitch match functions; this may indicate a difference in development of place pitch perception as well as difficulty in task learning. To quantify the number of pitch match successes and failures, task completion was defined as having a monotonic pitch match function with clear 0% and 100% points within the range of comparison stimuli (example shown in Fig. 3B) [29]. Specifically, if the pitch match function did not reach 0 or did not reach 1 (Fig. 3C), this indicated an inability to consistently rank a comparison stimulus as lower and higher in pitch than the reference, respectively, and thus an inability to pitch match within the range of comparison stimuli presented. This strict data inclusion criterion is necessary to ensure the accuracy of pitch match data, as it has been shown that with unclear pitch percepts such as those through a CI, pitch matches are highly susceptible to range effects [30]. In listeners with hearing loss, this range was sometimes limited by available residual hearing or electrodes. In NH cases such as in Fig. 3C, it is also possible that a wider frequency range could have been selected and successful pitch matching achieved. Task failures do not always indicate an inability to pitch match, but can also demonstrate the exclusion of unusable data points due to limited comparison stimulus ranges. Task completion failures were excluded from further data analyses.

### Conversions for Comparisons Across Groups and CI Manufacturers

In order to compare pitch match and fusion results across CI manufacturers and devices with different inter-electrode distances, fusion ranges and pitch match ranges estimated for BK subjects were converted from units of electrodes to units of millimeters (measured between the centers of adjacent electrodes) using the following inter-electrode distances based on physical electrode spacing: 2.4 mm for the MED-EL Standard array, 0.75 mm for Cochlear CI24M, and variable distances between 0.4 and 0.81 for Cochlear CI24R/CI24RE/CI512 and between 0.85 and 0.95 for Cochlear CI422 arrays. In order to avoid creating artificial differences in fusion ranges within the bilateral CI group, with different internal electrode arrays and inter-electrode spacing by manufacturer, maximum fusion ranges and pitch match ranges were defined to be 10.92 mm (the length of the shortest array in the data set) for all bilateral CI subjects.

In order to compare results from the BK group, in units of millimeters, to the results from other groups, the scale in millimeters was approximated to octaves based on the observed mostly linear relationship of cochlear place to log frequency above the 1000 Hz cochlear place for both the organ of Corti and spiral ganglion maps (~ 1 octave per 5 mm) [31, 32].

### Statistical Methods

Data from the fusion range and pitch match tasks were converted to octaves. For participants in the HK, NK, and CK groups, fusion range (FR) was calculated by taking the log of the higher frequency 50% point divided by the lower frequency 50% point, all divided by log(2): log(high/low)/log(2). The pitch match bandwidth (PMBW) for these participants was calculated similarly by taking the log of the 75% point divided by the 25% point, all divided by log(2): log(PM75/PM25)/log(2). For BK participants, a custom function was written in MATLAB to convert FR and PMBW values to mm. These values were divided by 5 to convert to octaves for comparison with the acoustic frequency ranges of the other groups (Section IIE). For participants who had multiple complete trials in a given year, the mean FR and PMBW values were calculated for the year. PMBW data were natural-log-transformed as the original data was highly skewed. Task completion was assessed as well, and PM and FR measurements were only included if the task was completed successfully.

Task completion with increasing age over time was investigated using mixed-effects logistic regression with a random intercept by participants. Task performance was examined using linear mixed-effects models with a random intercept. All models included a group-by-age interaction, as well as the main effects of group and age. Because exploratory analyses showed that baseline task completion and performance tended to be worse than follow-up years, an indicator for whether or not the data came from a baseline visit was also included in the models. There were seven pairs of siblings enrolled in the study. Sensitivity analyses were conducted by adding family as an additional level of clustering in the models.

Data management and analyses were conducted using Stata 16.1. Final figures were generated using SAS Software 9.4.

## Results

**Fig. 4:**
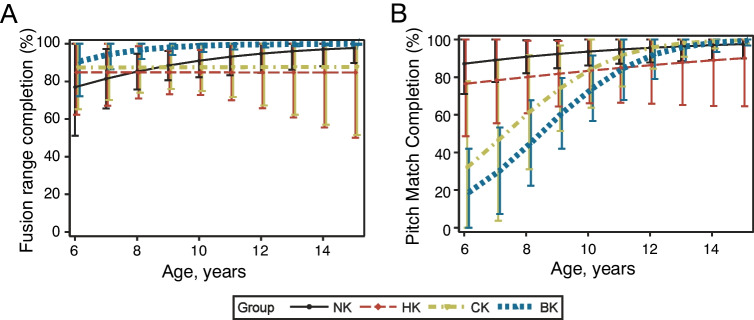
Task completion rates for the four groups for fusion range (**A**) and pitch matching (**B**). Mixed-effects logistic regression models included age, group, an age-by-group interaction, and an indicator for whether the data came from a baseline visit. Error bars indicate 95% confidence intervals

Figure 4 shows the task completion rates by group for the fusion range task (Fig. 4A) and the pitch match task (Fig. 4B). For fusion range task completion, there was a main effect of group (*p* = 0.0197), but not age (*p* = 0.320), and no significant group-by-age interaction (*p* = 0.6464). The effect of group appears to be driven by BK participants having the highest completion rates overall (Fig. 4A). For pitch match task completion, the main effects of age (*p* < 0.001) and group (*p* = 0.0048) were significant, but there was no significant group-by-age interaction (*p* = 0.1965). The model estimates in Fig. 4B show that the age effect is due to the improvement of task completion over time. Group differences appear to be driven by the CK and BK groups having lower task completion, especially at earlier ages.

**Fig. 5:**
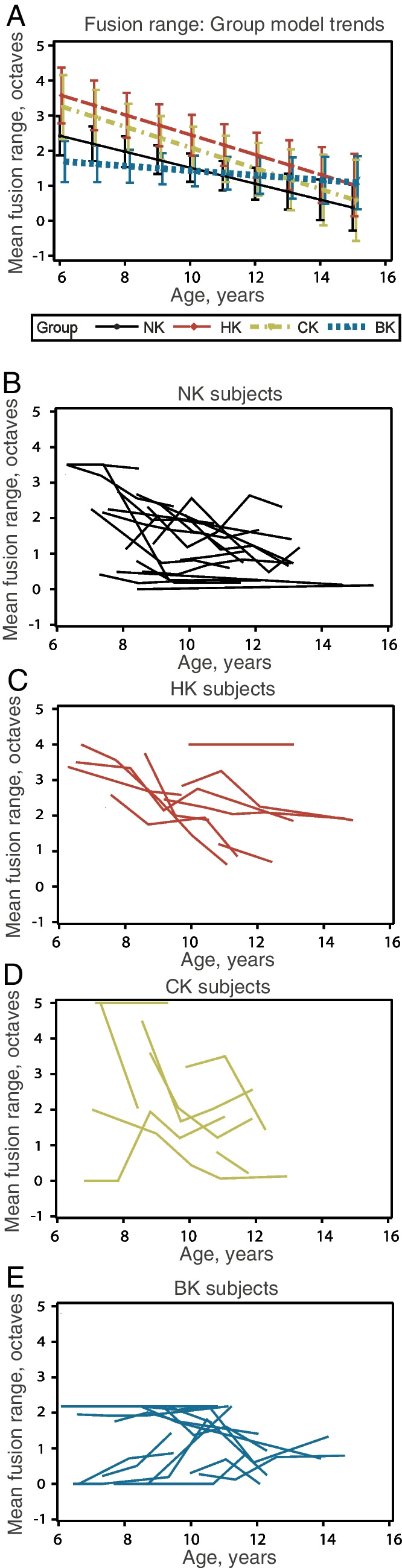
Longitudinal changes in fusion range with age. **A** Group model results. Error bars indicate 95% confidence intervals.** B–E** Individual results by subject, for NK (**B**), HK (**C**), CK (**D**), and BK (**E**) groups

Figure 5 shows the longitudinal changes in the fusion range with age. There was a significant group-by-age interaction (*p* = 0.0204), as well as significant main effects of age (*p* < 0.001) and group (*p* = 0.0145). The interaction appears to be driven by BK participants having fairly stable performance as a group with increasing age, while the other three groups demonstrate decreasing FR with increasing age (Fig. 5A). Figure 5B–E display FR performance versus age for individual participants in the four groups.

**Fig. 6:**
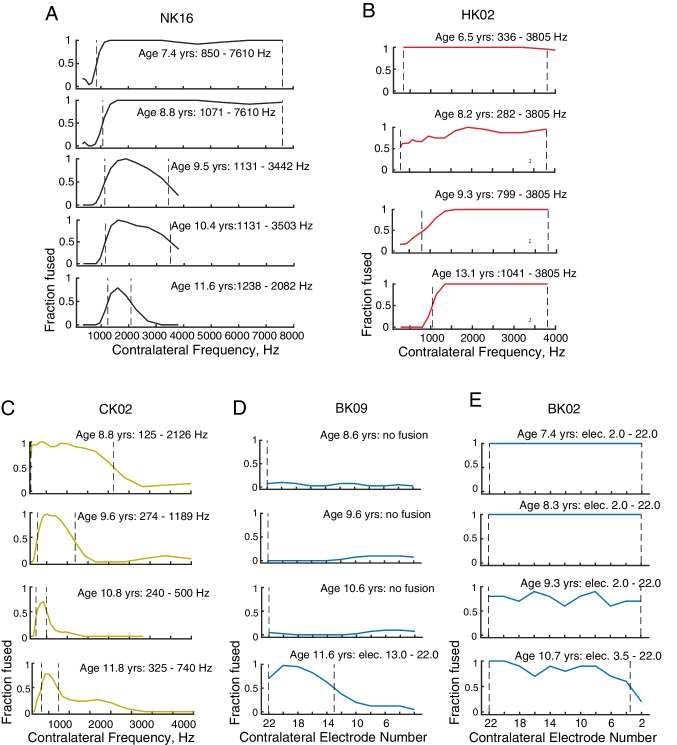
Representative examples of longitudinal changes in fusion functions and fusion range with age. Fusion functions are shown with descending rows indicating later years in the study. Age at the time of testing is given for each year, along with fusion ranges.** A** NK subject with fusion functions becoming narrower on both low- and high-frequency sides with age. **B** HK subject with fusion functions narrowing asymmetrically (more on the low-frequency side) with age. **C** CK subject with fusion functions narrowing on both sides with age. **D** BK subject with fusion range decreasing slightly with age. **E** BK subject with fusion range increasing slightly with age

These trends can be seen more clearly for individual fusion functions from representative subjects from each group in Fig. 6. The NK subject shows the greatest decrease in fusion range with age over all the groups, with narrowing of the fusion function from both the low- and high-frequency sides, starting from a fusion range of 850–7610 Hz (3.2 octaves) at age 7.4 years in the first year and narrowing progressively to a fusion range of 1238–2082 Hz (0.75 octaves) by age 11.6 years (Fig. 6A). This symmetric narrowing is apparent for most NK subjects (Suppl. Figure 2).

The HK subject (HK02) shows some decrease in the fusion range with age, driven mostly by narrowing of the fusion function from the low-frequency side, starting from a fusion range of 336–3805 Hz (3.5 octaves; the entire range of frequencies tested) at age 6.5 years in year 1 and narrowing to a fusion range of 1041–3805 Hz (1.9 octaves) by age 13.1 years in the final year (Fig. 6B). This lack of change on the upper frequency side is evident for most HK subjects (Suppl. Figure 3), though some subjects with better acoustic thresholds (HK03) or hearing asymmetry (HK09, HK13, HK14) showed some mild narrowing on the upper frequency boundary.

The CK subject (CK02) shows a decrease in fusion range with age more similar to the NK subject, with narrowing of the fusion function from both low- and high-frequency sides, starting from a fusion range of 125–2126 Hz (4.1 octaves) at age 8.8 years in year 1 and progressing to a fusion range of 325–740 Hz (1.2 octaves) by age 11.8 years (Fig. 6C). This trend is apparent for most CK subjects (Suppl. Figure 4). It should also be noted that those subjects who lost some hearing over the course of the study (HK01, HK10, HK13, CK01, CK08) did not show different trends from the other subjects (Suppl. Figure 3–4).

Interestingly, fusion ranges in the BK group did not change consistently with age, with individual participants showing no fusion from ages 8.6 to 10.6 years, followed by an increase at age 11.6 years (BK09; Fig. 6D), broad fusion and little to no change between the ages of 7.4 to 10.7 years (BK02, Fig. 6E), or a decrease (e.g., BK08, BK12 in Suppl. Figure 5). This variation is evident for the individual data (Suppl. Figure 5), and is consistent with the lack of overall significant change with age for the group.

**Fig. 7:**
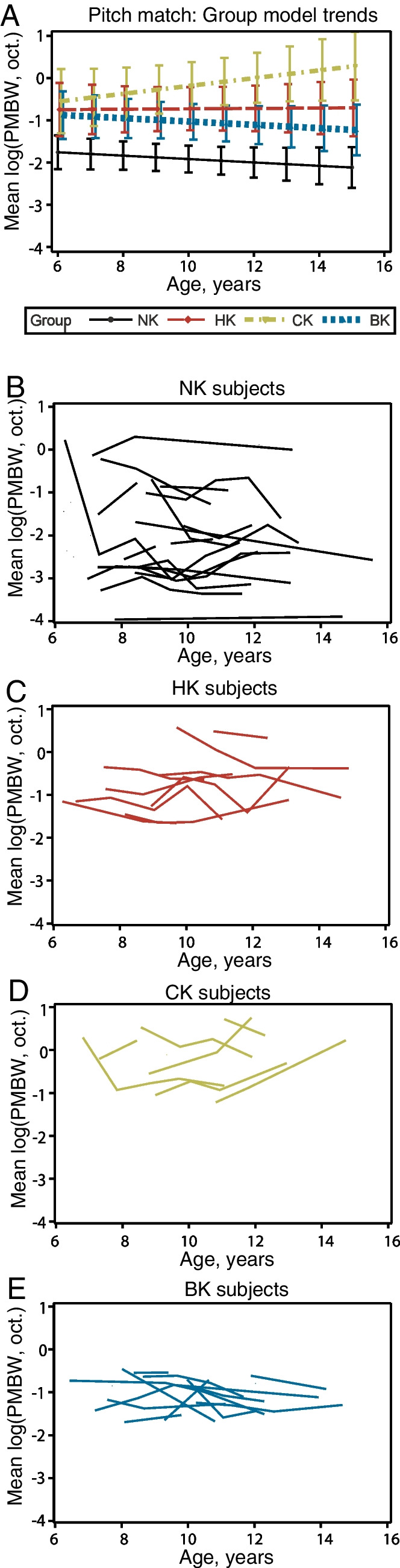
Longitudinal changes in pitch match range with age. Pitch match range is plotted as log(bandwidth in octaves), so that so that − 1 corresponds to 0.367 octaves, − 2 to 0.135 octaves, − 2.3 to 0.1 octaves, and so on. Error bars indicate 95% confidence intervals.** A** Group model results. **B–E** Individual results by subject, for NK (**B**), HK (**C**), CK (**D**), and BK (**E**) groups

Figure 7 shows the longitudinal changes in pitch match bandwidth (log-transformed values) with age. For pitch match bandwidth, there was a significant main effect of group (*p* < 0.0001), with no main effect of age (*p* = 0.647) or significant interaction between group and age (*p* = 0.2374). NK participants had the narrowest pitch match bandwidth, indicating sharper frequency tuning, followed by BK, HK, and CK participants (Fig. 7A). Figure 7B–E show the individual participant trajectories with age in each of the four groups. Note that the natural log transformation means that a value of − 1 corresponds to 0.367 octaves, − 2 to 0.135 octaves, − 2.3 to 0.1 octaves, and so on. Compared to fusion ranges for each group, these pitch match bandwidths are much narrower at scales of fractions of an octave compared to fusion ranges closer to the order of octaves.

The sensitivity analyses including clustering based on family did not change any study conclusions, and model estimates were not substantially different.

## Discussion

As this study was conducted in young children, it was important to conduct analyses to check that data met the criteria for inclusion, i.e., “successful task completion,” with results indicating that the child understood the task and was able to consistently and reliably attend to the task. Task completion for the fusion range task was generally stable with age, indicating that all groups were able to conduct the task from the start. In contrast, for the pitch match task, the task completion rate improved with age for children with CIs, particularly those in the BK group, as they had difficulty completing the pitch match task as well as within-ear pitch ranking at younger ages. This suggests a potential advantage of natural acoustic hearing experience in earlier development of place pitch perception, at least in the cochlear place domain. However, it should be noted that the methods of this experiment were restrictive in only allowing cues for pitch based on electrode cochlear place, and eliminated temporal envelope cues, in order for performance to be interpreted as derived solely based on place cues. Hence, the results here do not rule out that children with CIs had the ability to perceive pitch early on, i.e., use temporal envelope cues rather than place cues for pitch perception in their everyday CI listening configurations which transmit temporal envelope as well as place cues.

Interaural pitch match range did not change significantly with age for any of the groups for those cases where children were able to complete the task. This finding suggests that interaural pitch discrimination abilities do not improve between the ages of 6 and 14 years, once minimal pitch perception based on place cues is established. Although previous studies have shown improvement in other types of perception during development, such as monaural pitch ranking, frequency discrimination, amplitude modulation detection, and temporal integration [1, 2, 33], those studies showed differences mainly between 5 and 8 years of age. Our dataset encompassed a higher range of 6–14 years of age, when these basic perceptual abilities are likely near adult-like. The average interaural pitch match ranges on the order of 0.135 octaves in children with NH were also similar to the average 0.16 ± 0.25 octaves found in NH adults in our previous study [19].

Fusion range, on the other hand, was broad initially and decreased significantly with age for children with NH, bilateral HAs, and bimodal CIs, indicating that binaural pitch fusion sharpens for these groups during development between the ages of 6 and 14 years. As interaural pitch discrimination did not change with age, changes in pitch discrimination do not explain the observed changes in fusion range with age. This refinement of binaural fusion from early childhood to adolescence is consistent with improvement seen for binaural fusion of co-modulated low- and high-pass stimuli [34, 35]. These findings are also consistent with the refinement in this age range observed across other sensory modalities, such as visual (binocular) and multisensory integration [36, 37]. Studies in the visual system suggest that young children between 6 and 8 years of age process binocularly disparate cues differently from older children and adults [36]. A study in children with normal vision revealed that dichoptic integration of different inputs to the two eyes and binocular summation both decrease during development, suggesting that binocular integration is still developing in children up to 14 years of age [37]. Similarly, multisensory integration does not mature until 8 years of age [38]. This suggests a parallel for binaural integration to also be developing in this age range. In particular, the corpus callosum, which is topographically organized and attains 90% of its specificity by 11 years of age, may reflect a common trajectory for pruning of connections that mediate bilateral sensory integration [39].

However, changes were greater for children with NH, bilateral HAs, and bimodal CIs, than for children with bilateral CIs. These findings suggest that for those with hearing loss, certain hearing device combinations, particularly the bimodal CI configuration, may promote more beneficial sharpening of binaural fusion during development than other device combinations. However, it should be noted that the bilateral CI group differed from the others in the presence of three sub-groups, those who started out with zero to narrow fusion and showed increases in fusion with age, those who started out with broad fusion and showed decreases in fusion with age, and those who showed no change, leading to a lack of consistent group trend.

In adult bilateral HA users, it has been shown that longer durations of hearing loss and/or early onset of hearing loss and HA use are associated with broader fusion [10]. During development and learning, plasticity is mediated by various processes including synaptic pruning of neural connections mediated by spike-timing dependent plasticity (STDP), such that synapses are strengthened by coincident inputs and weakened by asynchronous inputs. Based on the correlations with long durations and early onset of hearing loss, Reiss et al. [10] proposed that in bilateral HA users, amplification and the resulting spread of excitation could interfere with synaptic pruning that sharpens binaural fusion by increasing the prevalence of coincident spikes across different frequencies across ears, thus preventing neural pruning of such connections. This concept of reduced neural pruning may apply to children with bilateral HAs in this study, particularly on the high-frequency side with greater hearing loss, where less reduction in fusion was seen with increasing age compared to the children with NH. This would be in contrast with the normal synaptic pruning mediated by normal acoustic thresholds, in which coincident spikes would only occur for and strengthen inputs from matched frequencies across ears at lower sound levels and in the absence of abnormal spread of excitation, whereas the lack of coincident spikes for different frequency inputs across ears would lead to pruning of those connections. For bilateral CI users, there is a large spread of current and similar spiking patterns induced by the constant rate stimulation from the cochlear implant, similar to that experienced by bilateral HA users. For bimodal CI users, on the other hand, although there might be a large spread of current in the CI ear and spread of excitation in the HA ear, the differences in spiking patterns between electric and acoustic stimulation may reduce the prevalence of coincident spikes for binaural inputs for frequencies outside of the mapped frequency range for each electrode.

As noted, a subset of children with bilateral CIs had extremely narrow or no fusion to start with, also demonstrated in Hartling et al. [19]. This differs from the broader fusion observed in bilaterally implanted adults [12, 13]. This lack of fusion in children, at least for same-numbered electrodes, was also observed in a previous study of children with bilateral CIs [40]. One possibility is that because most of these children with bilateral CIs are born deaf, just as lack of early acoustic input hinders the development of tonotopic maps [20–22], the lack of early *bilateral* input may hinder the initial formation of those inter-hemispheric connections, regardless of whether the input is bilateral natural acoustic stimulation, bilateral electric stimulation, or a mix of acoustic and electric stimulation across ears, i.e., bimodal stimulation. Another possibility is that bilateral electric stimulation alone may not be adequate (or very slow compared to acoustic hearing) for driving STDP, due to the lack of synchronization of timing of stimulation from the bilateral CI processors combined with the strong entrainment observed with electric stimulation. More research is needed to determine the factors underlying the lack of binaural fusion in children implanted with bilateral CIs early in life.

However, it is not yet clear if there is a critical time window for such development to benefit from bimodal CI use, i.e., whether children with bilateral HAs could get a CI later in life and still be able to benefit from and further sharpen fusion on the high-frequency side. Further research is needed in adolescents and young adults in order to better understand the effects of hearing device combination on the refinement of binaural fusion later in life. Such studies will be of clinical relevance for guiding patients on when to transition from bilateral HAs to bimodal CI use and similarly for when to transition from bimodal CI use to bilateral CI use, while also accounting for changes in residual hearing and the continued ability to use HA(s).

One limitation is that it is possible that improved conceptual understanding or task learning could have contributed to the observed changes. For the interaural pitch discrimination task, the improvement in task completion rate certainly supports this interpretation for children who use bimodal and bilateral CIs, as an alternative to improved perception of pitch in itself. For the fusion range task, this interpretation seems less likely given the gradual rather than sudden changes seen in the fusion range with age, as shown for the example participants in Fig. 6.

Overall, these findings indicate a fundamental change in binaural integration of frequency information during pre-adolescent development, even for children with NH. The initial binaural fusion ranges were surprisingly broad for NH children, with many starting at 2–4 octaves, and much broader than for NH adults who typically experience fusion on the order of 0.1–0.2 octaves [19]. Immature binaural fusion may be one underlying mechanism for the greater difficulty that children have in understanding speech in noisy environments, compared to adults. In adults, broad binaural fusion has been shown to be negatively correlated with the ability to benefit from fundamental frequency (F0) differences for understanding speech in the presence of competing talkers, either for two-talker scenarios or realistic multi-talker environments [15–17]. Consistent with these effects of broad binaural fusion, NH children do not get as much benefit from F0 difference cues as NH adults [41] and have greater difficulties than NH adults with understanding speech in the presence of competing talkers [42, 43]. A practical implication is that if children have greater difficulties with speech perception in talker noise due to immature/broad binaural fusion, quiet classroom environments will be especially beneficial for learning.

Finally, the finding of broad binaural fusion in children with NH expands our options for studying the neural and anatomical correlates of binaural fusion in both animal and human models, with potential application for targeted treatment of broad binaural fusion in adults with hearing loss. Neurophysiological correlates and neuroanatomical changes during pre-adolescent development can be studied in young animals with NH without the need to develop complex animal models of chronic hearing loss, HAs, and/or CIs to induce broad binaural fusion. Electrophysiological measures and functional magnetic resonance imaging (fMRI) are more feasible in children with NH than in those with HAs or CIs, and longitudinal studies in the pre-adolescent age range in particular may shed light on where and how binaural fusion is mediated in the brain.

## Supplementary Information

Below is the link to the electronic supplementary material.

Supplementary Figure 1. Example comparison of interaural pitch matching results from simultaneous and sequential tone presentation in a normal-hearing participant, NK04. Simultaneous and sequential pitch matching results are shown from the fusion range task (light blue dotted curve) and interaural pitch matching task (solid dark blue curve), respectively. The fusion function obtained from the fusion range task is also shown in green with square symbols. The two rows represent years 1 and 2 of data collection. In both cases, the pitch match function slope is steeper and the range is narrower for the sequential (dark blue) than simultaneous (light blue) tone presentation. (JPG 176 KB)

Supplementary Figure 2. Individual fusion range changes with age split by upper and lower boundaries relative to the reference frequency for the NK group. Reductions in fusion range are apparent from both directions. (JPG 203 KB)

Supplementary Figure 3. Individual fusion range changes with age split by upper and lower boundaries relative to the reference frequency for the HK group. HK subjects have asymmetric changes in fusion range, with much less reduction on the upper frequency side compared to NK subjects (compare with Suppl. Fig. 2). (JPG 134 KB)

Supplementary Figure 4. Individual fusion range changes with age split by upper and lower boundaries relative to a reference frequency of 750 Hz for the CK group. For this visualization, reference frequency is set to the center of the frequency-to-electrode allocation for the reference electrode in the CI ear, and is not necessarily in the fusion range for all subjects, so that some lower and upper fusion range boundaries are above and below the reference, respectively. (JPG 133 KB)

Supplementary Figure 5. Individual fusion range changes with age split by upper and lower boundaries relative to the reference electrode for the BK group. This reference is not necessarily centered in the fusion range for all subjects, so that some lower and upper fusion range boundaries are above and below the reference, respectively. (JPG 168 KB)

## Data Availability

The data will be available from the authors upon reasonable request.

## Code Availability

Code for child-friendly programs and cochlear implant stimulation via research interfaces written in MATLAB and Visual C will be available from the authors upon reasonable request.
