# Supplementary figures and images for: Binaural Fusion Sharpens on a Scale of Octaves During Pre-adolescence in Children with Normal Hearing, Hearing Aids, and Bimodal Cochlear Implants, but not Bilateral Cochlear Implants
Source: J Assoc Res Otolaryngol. 2025 Feb 6;26(1):93–109. doi: 10.1007/s10162-025-00975-4 (PMC11861472; doi:10.1007/s10162-025-00975-4)

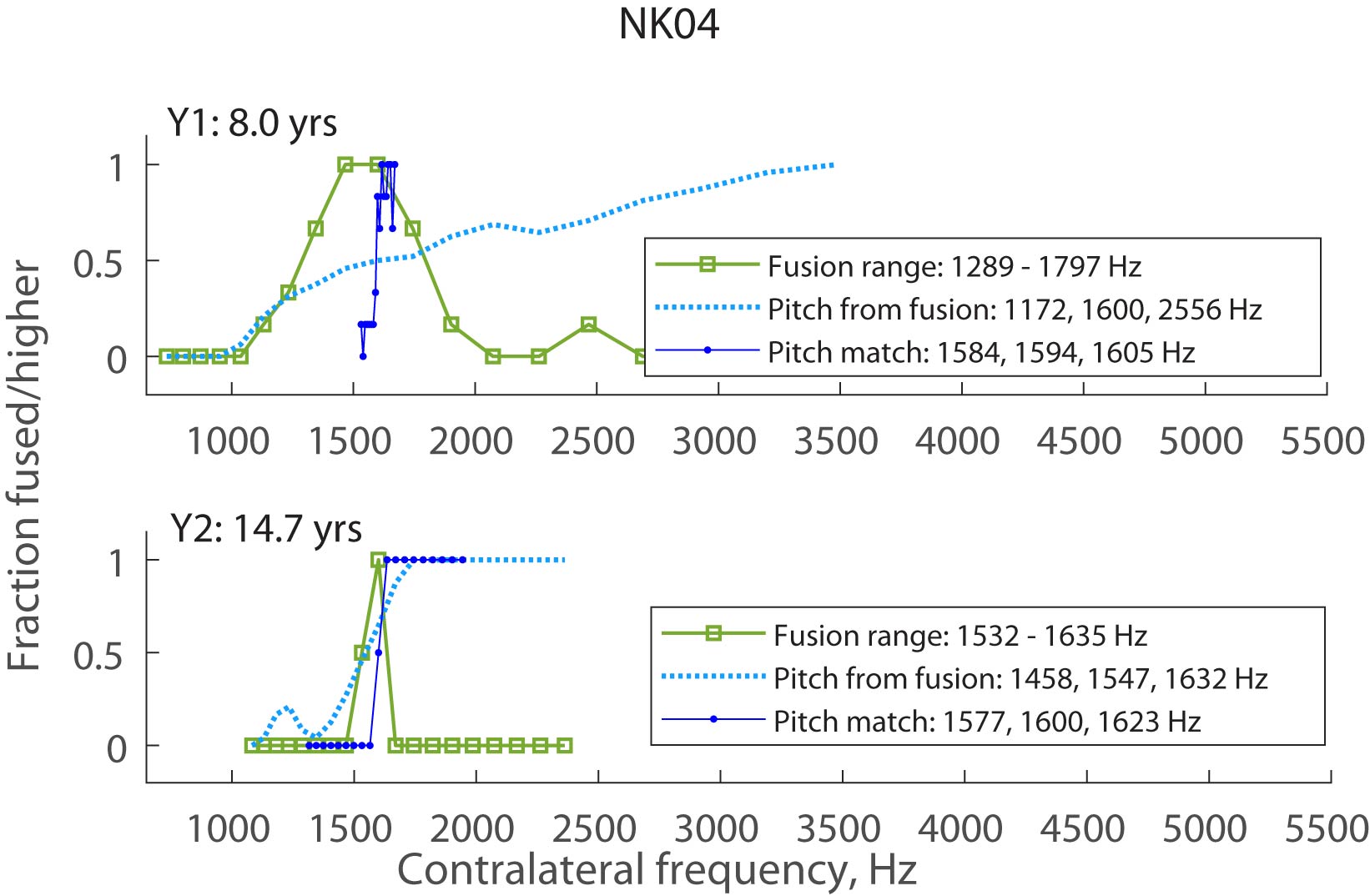

Supplement: Supplementary file 1 — Supplementary Figure 1. Example comparison of interaural pitch matching results from simultaneous and sequential tone presentation in a normal-hearing participant, NK04. Simultaneous and sequential pitch matching results are shown from the fusion range task (light blue dotted curve) and interaural pitch matching task (solid dark blue curve), respectively. The fusion function obtained from the fusion range task is also shown in green with square symbols. The two rows represent years 1 and 2 of data collection. In both cases, the pitch match function slope is steeper and the range is narrower for the sequential (dark blue) than simultaneous (light blue) tone presentation. (JPG 176 KB) [file 10162_2025_975_MOESM1_ESM.jpg]

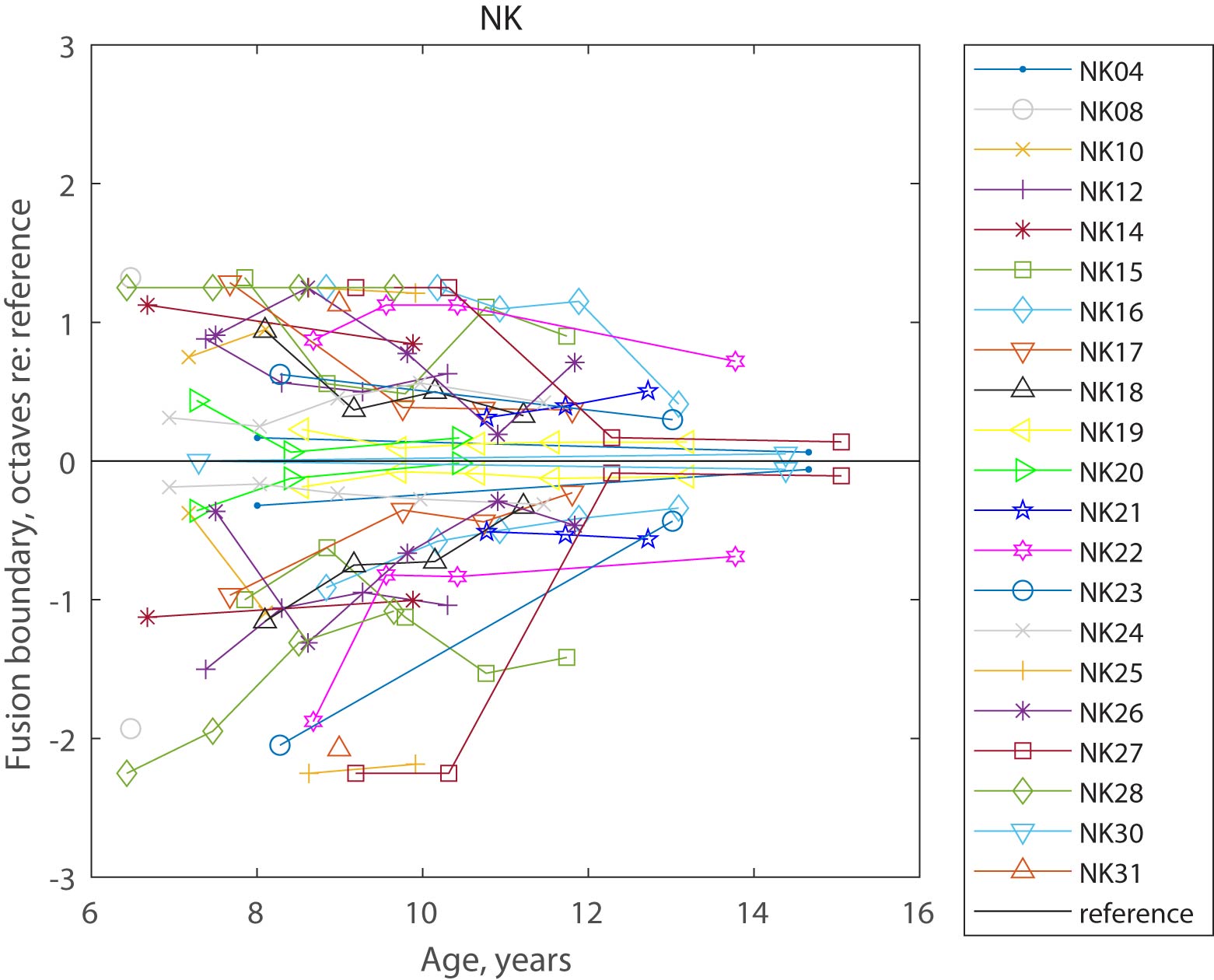

Supplement: Supplementary file 2 — Supplementary Figure 2. Individual fusion range changes with age split by upper and lower boundaries relative to the reference frequency for the NK group. Reductions in fusion range are apparent from both directions. (JPG 203 KB) [file 10162_2025_975_MOESM2_ESM.jpg]

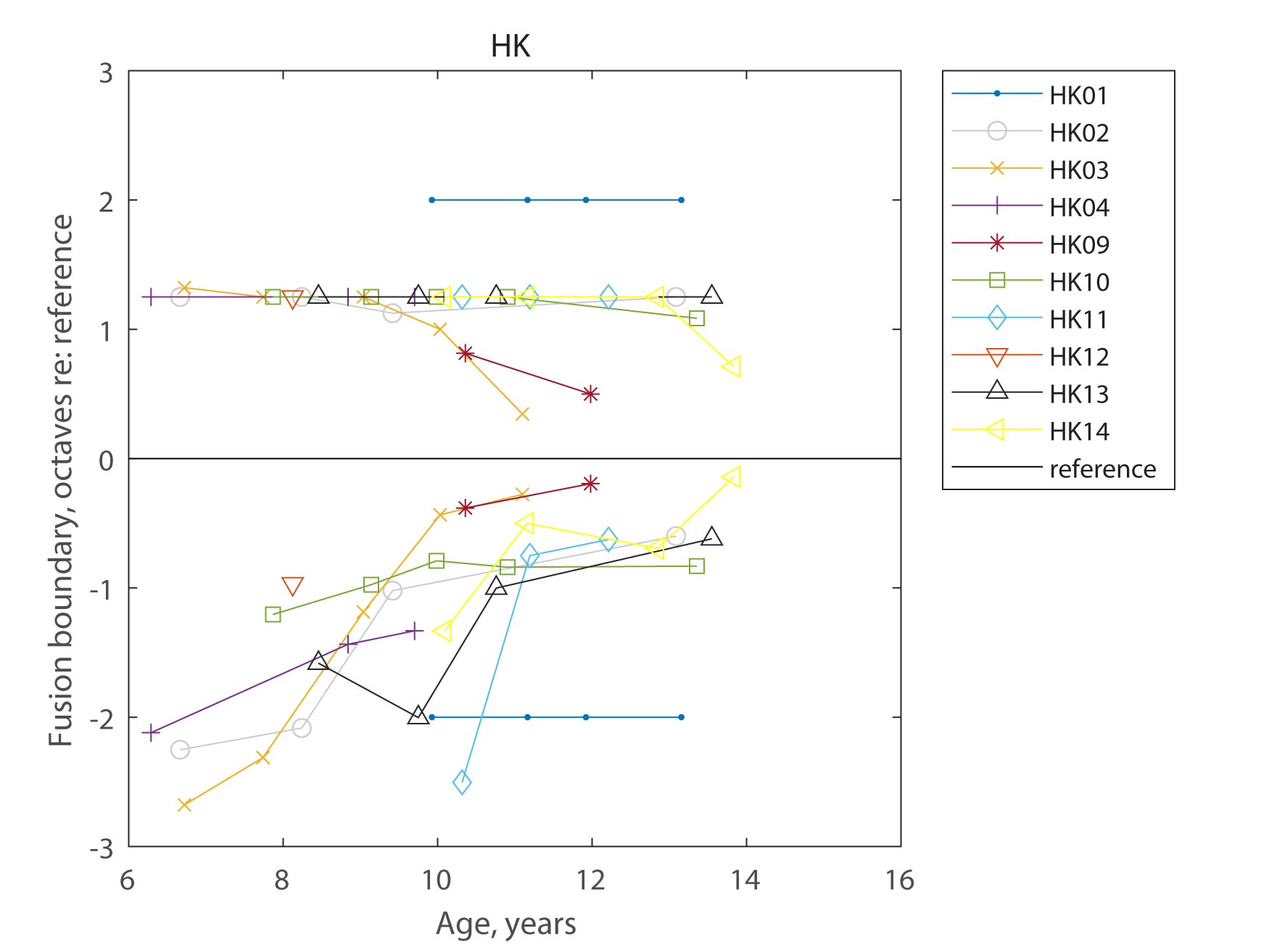

Supplement: Supplementary file 3 — Supplementary Figure 3. Individual fusion range changes with age split by upper and lower boundaries relative to the reference frequency for the HK group. HK subjects have asymmetric changes in fusion range, with much less reduction on the upper frequency side compared to NK subjects (compare with Suppl. Fig. 2). (JPG 134 KB) [file 10162_2025_975_MOESM3_ESM.jpg]

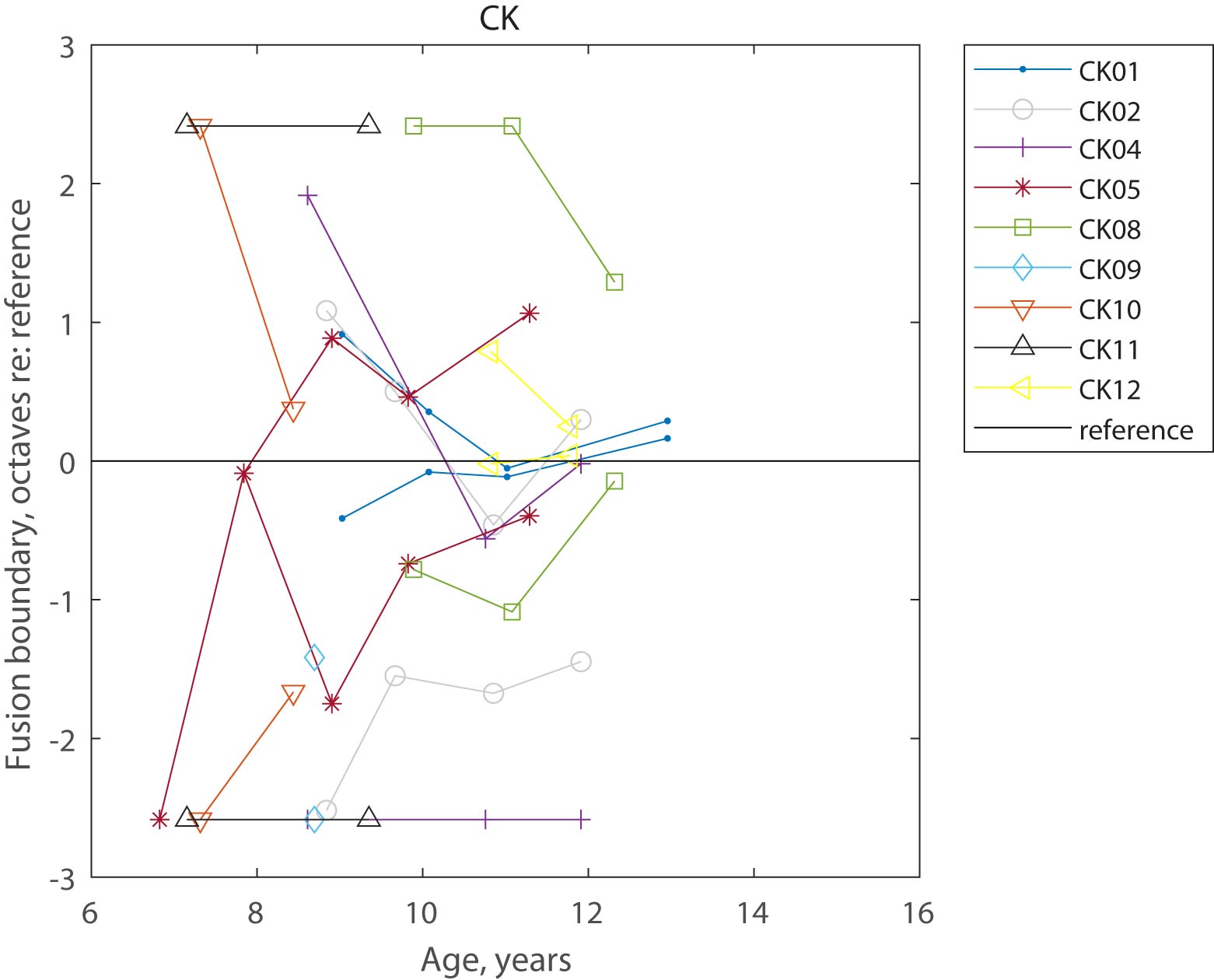

Supplement: Supplementary file 4 — Supplementary Figure 4. Individual fusion range changes with age split by upper and lower boundaries relative to a reference frequency of 750 Hz for the CK group. For this visualization, reference frequency is set to the center of the frequency-to-electrode allocation for the reference electrode in the CI ear, and is not necessarily in the fusion range for all subjects, so that some lower and upper fusion range boundaries are above and below the reference, respectively. (JPG 133 KB) [file 10162_2025_975_MOESM4_ESM.jpg]

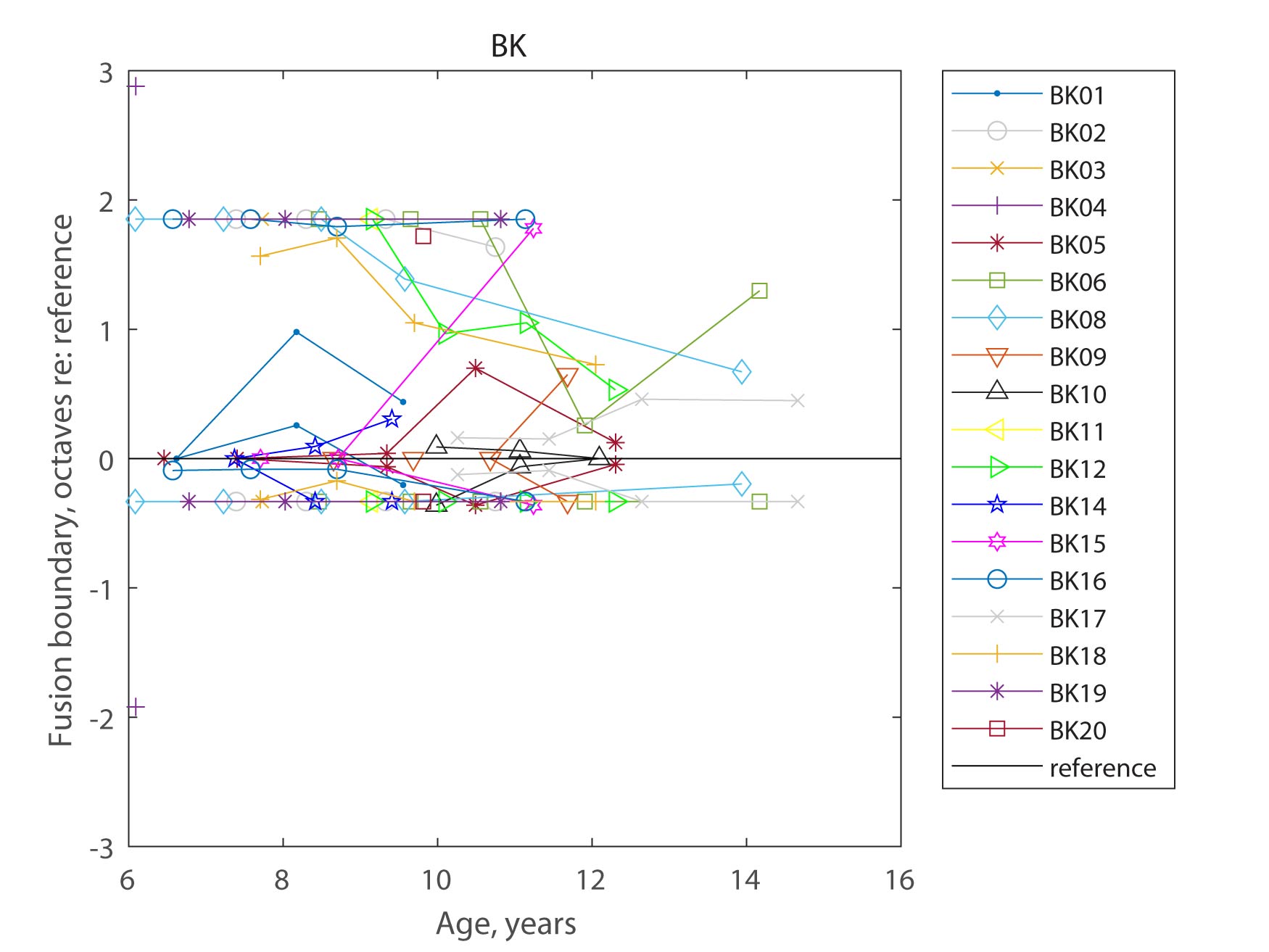

Supplement: Supplementary file 5 — Supplementary Figure 5. Individual fusion range changes with age split by upper and lower boundaries relative to the reference electrode for the BK group. This reference is not necessarily centered in the fusion range for all subjects, so that some lower and upper fusion range boundaries are above and below the reference, respectively. (JPG 168 KB) [file 10162_2025_975_MOESM5_ESM.jpg]
